# Associations between prenatal alcohol exposure and parent‐reported sleep disturbances in 10,336 adolescents: an Adolescent Brain Cognitive Development study

**DOI:** 10.1111/jcpp.70165

**Published:** 2026-05-21

**Authors:** Emma K. Devine, ReJoyce Green, Rachel Visontay, Hollie Byrne, Julia Riches, Elizabeth J. Elliott, Nicola C. Newton, Lindsay M. Squeglia, Louise Mewton, Lexine A. Stapinski

**Affiliations:** ^1^ The Matilda Centre for Research in Mental Health and Substance Use The University of Sydney Sydney NSW Australia; ^2^ Department of Psychiatry and Behavioral Sciences Medical University of South Carolina Charleston SC USA; ^3^ Specialty of Child and Adolescent Health, Faculty of Medicine and Health The University of Sydney Sydney NSW Australia; ^4^ The Sydney Children's Hospitals Network Sydney NSW Australia

**Keywords:** Sleep disturbances, prenatal alcohol exposure, adolescence

## Abstract

**Background:**

This study investigated the associations between prenatal alcohol exposure (PAE), including low and moderate levels of exposure, and parent‐reported sleep disturbances during adolescence. This is an area that remains understudied despite evidence linking PAE, particularly heavy PAE, to poor sleep in younger children and the growing recognition of harms associated with low levels of PAE.

**Methods:**

Participants were 10,336 adolescents (aged 12–13) from the fourth assessment wave of the Adolescent Brain Cognitive Development Study. Cross‐sectional generalised linear mixed models and generalised additive mixed models were used to assess the impact of prenatal alcohol exposure, conceptualised as the presence or absence of PAE, total drinks consumed during pregnancy (i.e. dose) and patterns of PAE (i.e. abstainers, light reducing, light stable, heavy reducing), on parent‐reported adolescent sleep disturbances, while controlling for important birth related, environmental and medical factors.

**Results:**

Adolescents with any PAE experienced worse overall parent‐reported sleep disturbances compared to those without, with sleep–wake transitions, excessive somnolence and sleep breathing being the domains most impacted. There was limited support for a dose–response relationship between low‐level PAE and sleep problems in adolescence. However, those with a pattern of PAE before knowledge of pregnancy, compared to abstainers, experienced greater problems with sleep–wake transitions and sleep breathing.

**Conclusions:**

These findings contribute to the growing evidence that there are no safe levels of alcohol consumption during pregnancy, as even low to moderate PAE negatively impacts adolescent sleep. Identifying sleep–wake transitions, excessive somnolence and sleep breathing as the most affected domains provides targets for both screening and intervention.

## Introduction

Healthy sleep is essential for child and adolescent development, with a vast literature highlighting the importance of sleep for neurological, physiological, cognitive, behavioural and emotional development (Agostini & Centofanti, 2024; Tarokh, Saletin, & Carskadon, 2016). Poor sleep, which includes sleep that is insufficient, inconsistent, not restorative, disrupted or poorly timed (Gariepy et al., 2020), has been associated with disordered development in each of these domains (Abel, Havekes, Saletin, & Walker, 2013; Beebe, 2011; Dickstein & Moldofsky, 1999; Orzech, Acebo, Seifer, Barker, & Carskadon, 2014; Perkinson‐Gloor, Lemola, & Grob, 2013; Shimizu, Zeringue, Erath, Hinnant, & El‐Sheikh, 2021).

Many factors can negatively impact sleep in children and adolescents, one of which is prenatal alcohol exposure (PAE). Globally, 9.8% of women consume alcohol during pregnancy, although this prevalence varies significantly by country and region, with the estimated prevalence of alcohol consumption during pregnancy in the United States being 14.8% (Popova, Lange, Probst, Gmel, & Rehm, 2017). Alcohol is teratogenic, affecting the growth and development and later function of the brain and other organs (Nava‐Ocampo, Velázquez‐Armenta, Brien, & Koren, 2004; Popova et al., 2023; van Faassen & Niemelä, 2011), with heavy PAE, particularly binge drinking, and PAE during periods of rapid foetal brain development posing the greatest risk (May et al., 2013; Petrelli, Weinberg, & Hicks, 2018).

Sleep problems are a commonly reported concern for children and adolescents with Foetal Alcohol Spectrum Disorder (FASD; Hanlon‐Dearman, Chen, & Olson, 2018), the most disabling potential consequence of PAE (Popova et al., 2023). Studies indicate that as many as 58%–85% of those with FASD (aged 4–18 years) experience sleep disturbances (Chen, Olson, Picciano, Starr, & Owens, 2012; Goril, Zalai, Scott, & Shapiro, 2016), which is higher than the 40% documented in typically developing children (Gradisar, Gardner, & Dohnt, 2011; Owens, Spirito, McGuinn, & Nobile, 2000). The type of sleep problems experienced varies, the most common being difficultyfalling asleep and staying asleep (Dylag et al., 2021; Hayes, Moritz, & Reid, 2020; Mughal, Hill, Joyce, & Dimitriou, 2020; O'Rourke et al., 2024; Wengel, Hanlon‐Dearman, & Fjeldsted, 2011).

Results of studies examining the impact of heavy PAE on sleep in children without a diagnosis of FASD align with those in children with a diagnosis of FASD. In a population‐based sample of infants, Alvik, Torgersen, Aalen, and Lindemann (2011) found that binge drinking at least once a week during the first 6 weeks of pregnancy strongly predicted sleep problems, conceptualised as short sleep durations and sleep interruptions, during infancy. In addition, alcohol use during pregnancy at >1 drink per day has been linked to increased rates of nightmares and sleep talking (Shang, Gau, & Soong, 2006), night wakings, parasomnias (Inkelis & Thomas, 2018) and overall sleep problems (Alvik et al., 2011; Harskamp‐van Ginkel et al., 2020) in childhood. These sleep difficulties negatively impact the individual's daytime functioning, further exacerbating their cognitive, psychological and behavioural impairments (Blackmer & Feinstein, 2016) and are strongly associated with poor caregiver well‐being and family quality of life (Hayes et al., 2020).

Although there is an established link between heavy PAE and FASD on offspring outcomes, less is known about the impact of low and moderate levels of alcohol consumption during pregnancy. Recently, Lees et al. (2020) undertook one of the most comprehensive examinations of the impact of PAE on offspring outcomes in a large sample of 9–10‐year‐old children, where those with PAE were exposed to, on average, 26.9 (*SD* = 24.5) drinks across gestation (i.e. <1 drink per week). They found that even low‐levels of PAE were associated with altered brain structure, and more psychological and behavioural problems, compared to those without PAE. This highlights the importance of examining the whole spectrum of PAE. To our knowledge, only one study has examined the impact of low and moderate, alongside heavy, PAE on offspring sleep outcomes. This longitudinal study (Chandler‐Mather, Occhipinti, Donovan, Shelton, & Dawe, 2021) followed 2‐year‐old children through to age 9 and found that heavy PAE, but not low or moderate PAE, was associated with more sleep problems, and that these problems persisted across childhood. Notably, this study focussed on childhood, and no studies have explored the impact of low and moderate PAE on offspring sleep outcomes during adolescence, a critical developmental period with unique environmental (i.e. school demands) and biological (i.e. changes to circadian processes) changes that impact sleep (Crabtree & Williams, 2009). As such, further research is required.

In the present study, we aim to examine the long‐term impacts of PAE on sleep outcomes in a large US sample (*N* = 10,336) of early adolescents (12–13‐year‐olds). PAE will be conceptualised in three ways, including the presence or absence of PAE, total drinks consumed during pregnancy (i.e. dose), and patterns of PAE (abstinence, light drinking before knowing of pregnancy, light drinking throughout pregnancy, heavy drinking before knowing of pregnancy). Sleep will be assessed using a validated parent‐report assessment tool that measures overall sleep problems and six subdomains (disorders of initiating and maintaining sleep, sleep breathing disorders, disorders of arousal, sleep–wake transition disorders, disorders of excessive somnolence and sleep hyperhidrosis). The use of the Adolescent Brain Cognitive Development (ABCD) study means we have access to a large cohort with extensive measures, allowing us to comprehensively assess PAE and sleep outcomes as well as control for potentially confounding factors, including birth related, environmental and medical factors. Taken together, this will allow for a rigorous examination of associations between PAE and sleep.

We tested three pre‐registered hypotheses (https://osf.io/sncw7/overview). First, given the established associations between PAE and sleep problems, we hypothesised that adolescents who were exposed to alcohol at any time during pregnancy would report higher levels of parent‐reported sleep problems, compared with adolescents without PAE. Second, given that heavy PAE is typically associated with worse sleep outcomes, we hypothesised that higher doses of PAE would be associated with more parent‐reported sleep problems during early adolescence. Third, aligned with prior evidence, we hypothesised that different patterns of PAE would be differentially associated with parent‐reported offspring sleep disorders.

## Methods

### Participants

The ABCD Study (Barch et al., 2018; Volkow et al., 2018) is a multi‐site (*N* = 21) longitudinal study tracking the biological and behavioural development of children from the ages of 9–10 through adolescence and into adulthood. We used data from the ABCD data release 5.1. PAE variables and covariates were derived from the baseline assessment wave (ages 9–10), whereas the sleep variables were obtained from the fourth assessment wave (ages 12–13, *N* = 10,336). Parents/caregivers provided signed informed consent and all participants gave assent. The ABCD protocol was approved by the centralised institutional review board (IRB) at the University of California, San Diego and by the IRBs at all sites.

### Measures

#### Sleep

Sleep outcomes were measured using the parent‐reported Sleep Disturbance Scale for Children (SDSC) (Bruni et al., 1996). The SDSC is a 26‐item questionnaire designed to assess multiple dimensions of childhood sleep, including: disorders of initiating and maintaining sleep, sleep breathing disorders, disorders of arousal, sleep–wake transition disorders, disorders of excessive somnolence and sleep hyperhidrosis (sweating). Parents respond on a 5‐point Likert scale (1 = Never, 5 = Always) about the degree to which their child experienced sleep disturbances over the past 6 months. A summed overall sleep disturbance score was also calculated. The SDSC performed well in a review of all childhood sleep scales (Lewandowski, Toliver‐Sokol, & Palermo, 2011) and has been shown to have good internal consistency (α = .71–.79) and test–retest reliability (*r* = .71) (Ağca, Görker, Turan, & Öztürk, 2021; Bruni et al., 1996; Lewandowski et al., 2011). In the present study, Cronbach's α values were .83 for overall sleep, and ranged from .36 to .81 for the sleep subscales (disorders of initiating and maintaining sleep α = .74, sleep breathing disorders α = .36, disorders of arousal α = .44, sleep–wake transition disorders α = .62, disorders of excessive somnolence α = .74, and sleep hyperhidrosis α = .81).

#### Prenatal alcohol exposure

PAE was measured using a modified version of the Developmental History Questionnaire (DHQ; Kessler et al., 2009; Merikangas, Avenevoli, Costello, Koretz, & Kessler, 2009). Mothers retrospectively reported on their/the child's biological mothers' alcohol use before and after knowledge of pregnancy (yes/no), the maximum number of drinks consumed on a single occasion before and after knowledge of pregnancy and the average number of drinks consumed per week during pregnancy before and after knowledge of pregnancy (see Table S1 in Appendix S1).

Using these questions, we calculated the following three variables according to methods outlined by Lees et al. (2020) for use in subsequent analyses: (a) a binary categorical variable reflecting any alcohol use at any time during pregnancy; (b) an estimate of the total number of drinks consumed during pregnancy which was winsorised at 1.5% to convert the most extreme outliers while otherwise maintaining the original data distribution; and (c) categories of common exposure patterns that have been previously applied to the ABCD data (Lees et al., 2020) and are based on an established classification of PAE (O'Leary et al., 2010). These categories include abstainers (abstinent throughout pregnancy), light reducers (light alcohol consumption before knowledge of pregnancy and abstinent after knowing of pregnancy), light stable users (light alcohol consumption throughout pregnancy) and heavier reducers (moderate, heavy or binge drinking before knowledge of pregnancy and abstinent or light drinking after knowing of pregnancy). Further detail on the calculation of these variables is provided in Appendix S1.

##### Fixed and random effect covariates

We adjusted for both fixed and random effect covariates. Included fixed covariates previously identified by Lees et al. (2020) were birth weight (in ounces); premature status (yes/no); week of maternal pregnancy knowledge; maternal age at time of birth; maternal use of other substances during pregnancy (yes/no) with tobacco, cannabis, cocaine and heroin each being included as separate variables; sex of adolescent at birth (female/male); adolescent race/ethnicity (White, Black, Hispanic, Asian, Other); adolescent age at time of assessment; maternal history of depression (yes/no); highest level of parental education (<high school diploma, high school diploma or equivalent, college, bachelor's degree, postgraduate degree). We also included the following fixed covariates that have been identified in the sleep literature as having evidence of a prior association with sleep outcomes: adolescent asthma (yes/no); adolescent brain injury (yes/no); adolescent cerebral palsy (yes/no); obesity (yes/no); history of paternal depression (yes/no); history of maternal and paternal anxiety (yes/no); adverse life events (total number of stressful life events perceived by the adolescent to be bad assessed using the Adverse Life Events Scale); family conflict (Family Conflict subscale of the Family Environment Scale; Moos & Moos, 1994); and caregiver acceptance (Child Report of Behaviour Inventory; Schaefer, 1965). To account for the clustered nature of the data (8,546 families and 21 data collection sites), random effects for family and site were included.

### Statistical analyses

#### Missing data

Missing data in the independent variables and covariates were addressed using multiple imputation. For the independent variables, we imputed at the item level (i.e. the items from the DHQ) rather than imputing the three PAE variables derived from these items, as recommended in the literature (Eekhout et al., 2014; Gottschall, West, & Enders, 2012). Specifically, we used the *mice* package (Van Buuren & Groothuis‐Oudshoorn, 2011) in R v4.4.1 (R Development Core Team, 2021) to derive multiply imputed datasets. In line with the rule of thumb whereby the number of imputations should be at least equal to the highest percent of missing data (Bodner, 2008; Graham, Olchowski, & Gilreath, 2007; White, Royston, & Wood, 2011; Wulff & Jeppesen, 2017), which in this study was 11.06% for the week of pregnancy knowledge variable, we derived 11 imputations. Missing data for the outcome variables was low (2.6%) and was addressed using full information maximum likelihood (FIML) in the statistical models. Further detail on missing data across all variables included in the present study can be found in Table 1 and in Table S2.

**Table 1:** Summary statistics for the prenatal alcohol exposure and parent‐reported sleep *outcomes* from the observed data prior to multiple imputation, split by whether the adolescents were unexposed or exposed to prenatal alcohol exposure

Variable	Unexposed adolescents	Adolescents with PAE	*p*‐Value^b^
	*N* = 7,550^a^	*N* = 2,582^a^	
Prenatal alcohol exposure (PAE) variables^c^			
Total drinks	0.00 (0.00)	26.47 (28.18)	<.001
Missing	1,043	464	
PAE patterns			
Abstainers	7,172 (100.0%)	3 (0.1%)^d^	<.001
Light stable use	0 (0.0%)	94 (4.3%)	
Light reducers	0 (0.0%)	1,278 (59.1%)	
Heavy reducing	0 (0.0%)	787 (36.4%)	
Missing	378	420	
Parent‐reported sleep variables			
Total sleep disorder score	35.70 (7.83)	36.75 (7.84)	<.001
Missing	188	76	
Disorder of initiating and maintaining sleep	12.15 (3.94)	12.40 (3.90)	.005
Missing	188	76	
Sleep breathing disorder	3.60 (1.08)	3.60 (1.02)	.786
Missing	188	76	
Disorder of arousal	3.25 (0.66)	3.27 (0.64)	.111
Missing	188	75	
Sleep–wake transition disorder	7.39 (2.10)	7.76 (2.32)	<.001
Missing	188	76	
Disorder of excessive somnolence	7.04 (2.58)	7.41 (2.71)	<.001
Missing	188	74	
Sleep hyperhidrosis	2.28 (0.89)	2.31 (0.89)	.136
Missing	188	76	

^a^ *n* (%); Mean (*SD*).

^b^ Welch two sample *t*‐test; Pearson's chi‐squared test.

^c^ 204 participants were missing data on whether they were unexposed or exposed to alcohol during pregnancy. The summary statistics presented above exclude those 204 participants. See supporting information file for more information.

^d^ Three participants reported having consumed alcohol before knowledge of pregnancy, 0 for the maximum number of drinks consumed on a single occasion before knowledge of pregnancy and 0 for the average number of drinks consumed per week before knowledge of pregnancy. When calculating the PAE patterns, this resulted in three participants in the PAE group meeting criteria for the abstainers PAE pattern.

#### Statistical analysis

A series of 21 models were run in R v4.4.1 (R Development Core Team, 2021). For the analyses focusing on the binomial (binary PAE; hypothesis 1) and multinomial PAE variables (PAE patterns; hypothesis 3), generalised linear mixed models (GLMMs) were conducted using the multiply imputed data and the *lme4* package (Bates, Mächler, Bolker, & Walker, 2014). Final estimates were obtained through aggregation procedures that follow Rubin's rules using the *parameters* (Lüdecke, Ben‐Shachar, Patil, & Makowski, 2020) and *miceadds* package (Robitzsch & Grund, 2024). For the dose‐dependent analyses focusing on the continuous (total drinks; hypothesis 2) PAE variable, we conducted a series of generalised additive mixed models (GAMMs) using the *mgcv* package (Wood, 2011). The observed data was used for the GAMMs as they produce F statistics and effective degrees of freedom (and their corresponding *p*‐values) which are not suitable for pooling using Rubin's rules following multiple imputation (Bolt et al., 2022). For all predictors, separate models were run for the seven sleep outcomes and each model included the covariates and random effects listed above. The multinomial analyses also included the winsorised total drinks variable as a covariate to get a more specific estimation of the effect of PAE timing, rather than volume, on sleep outcomes. To account for multiplicity, the false discovery rate (FDR; Benjamini & Hochberg, 1995) was used to correct for the 7 models with binary PAE, 7 models with continuous PAE and 21 models with PAE patterns, and the FDR adjusted p‐values are reported. Statistical significance was set at *p*_FDR_ < .05. The syntax is available on GitHub (https://github.com/emmakdevine/ABCD_Sleep_Paper).

### Sensitivity analyses

#### *E*‐values

For each statistically significant outcome observed from the generalised linear mixed models (those using the binary PAE and PAE patterns variables), we calculated *E*‐values for the estimate and for the limit of the confidence interval closest to the null to assess the sensitivity of the results to potential unmeasured confounding (VanderWeele & Ding, 2017). Larger *E*‐values were interpreted to mean that considerable unmeasured confounding would be needed to explain away an association, noting that what constitutes a large *E*‐value is context dependent (VanderWeele, Ding, & Mathur, 2019).

#### Inverse probability weighting

Inverse probability weighting (IPW) (Rosenbaum & Rubin, 1983) was used as a sensitivity analysis to further investigate the effect of PAE exposure (yes/no) on our outcomes of interest. IPW is an alternative to covariate adjustment in attempting to control for the effects of confounders and thus deliver causal insights (Visontay et al., 2024). Specifically, it re‐weights individuals to attempt to balance characteristics between the exposed and non‐exposed groups (Chesnaye et al., 2022). First, we fit a logistic regression model, regressing our binary PAE variable on all covariates included in the main analysis, from which we obtained propensity scores. Inverse probability weights were calculated from the propensity scores. Standardised mean differences between groups were calculated for all covariates before and after weighting to assess how well the groups are balanced (a standardised difference <10% is considered a negligible imbalance between groups). Kolmogorov–Smirnov statistics were used to ascertain that the distribution and variance were similar across groups (Kolmogorov–Smirnov statistics <.05 indicate better balance; Chesnaye et al., 2022). The main analyses for binary PAE (described above) were then repeated including probability weights and adjusting for covariates (i.e. a doubly robust analysis), with robust standard errors calculated to account for the uncertainty added by the propensity scores. The results were compared to those from the unweighted models. The R packages *WeightIt* (Greifer, 2023) and *MatchThem* (Pishgar, Greifer, Leyrat, & Stuart, 2020) were used for the analyses.

#### Binary outcomes

Model assumptions were assessed using the *performance* package in R (Lüdecke, Ben‐Shachar, Patil, Waggoner, & Makowski, 2021). Most of the models performed well, with some minor violations observed for homogeneity of variance, to which linear models with large sample sizes are relatively robust. However, three of the sleep subscales (sleep breathing disorder, disorders of arousal and sleep hyperhidrosis) exhibited more extreme violations of homogeneity of variance. To address this concern, we conducted an additional sensitivity analysis that conceptualised these three outcomes as binary variables, whereby participants who never reported experiencing any of the symptoms associated with the disorder were coded as 0 and those who reported any symptoms at any frequency were coded as 1. A series of nine generalised linear models were run (3 PAE predictors × 3 dichotomised SDSC subscales) which included the same covariates and random effects as the main models.

#### Deviations from the pre‐registration

The analysis presented here differed in five ways from the pre‐registered protocol. First, we used the ABCD 5.1 data release instead of the originally proposed 4.0 release, as a new wave of data became available after pre‐registration. This changed participants' age ranges from 11–12 to 12–13 years, which is better suited to investigating the impact of PAE on adolescent sleep outcomes. Second, paternal depression was included as a covariate in all models, given links between paternal depression and offspring sleep (Coles et al., 2022; Hall, Moynihan, Bhagat, & Wooldridge, 2017). Third, the total drinks variable used to assess a dose–response effect was included as an additional covariate in the models assessing patterns of PAE exposure (Hypothesis 3), to more accurately reflect the impact of patterns of PAE specifically on sleep outcomes. Fourth, due to computational constraints on bootstrapping multiply imputed data, we used robust standard errors to calculate the confidence intervals for the IPW sensitivity analyses. Fifth, we conducted an additional sensitivity analysis where three of the sleep subscales (sleep breathing disorder, disorders of arousal and sleep hyperhidrosis) were conceptualised as binary outcome variables to address violations of homogeneity of variance.

## Results

### Study sample

The summary statistics for the predictor and outcome variables included in this study, prior to multiple imputation, are presented in Table 1 (see Table S2 for the summary statistics for all variables included in the study). Of the 10,336 adolescents included in this study (*M*_age_ = 12.91, *SD*_age_ = 0.65, 47.5% female at birth), 25.5% (*N* = 2,582) had parent‐reported prenatal exposure to alcohol. The winsorised estimated total number of drinks consumed during pregnancy ranged from 0 to 116 and among those who had consumed alcohol, the mean number of drinks consumed throughout pregnancy was 26.47 (*SD* = 28.18). Regarding the common exposure patterns, most participants were classified in the abstinent group (*N* = 7,175), followed by light reducers (*N* = 1,278) and heavy reducers (*N* = 787) and the light stable group (*N* = 94).

### Binary prenatal alcohol exposure associations

Results of the covariate adjusted GLMMs using the multiply imputed data are provided in Table 2. Adolescents who were prenatally exposed to any alcohol experienced greater overall parent‐reported sleep disturbances (β = 0.517, 95% CI = 0.127–0.906, *p*_FDR_ = .021), compared to unexposed adolescents. Regarding the sleep disturbance subscales, adolescents exposed to any PAE exhibited more parent‐reported problems on the sleep–wake transition disorder scale (β = 0.210, 95% CI = 0.101–0.318, *p*_FDR_ = .001) and on the disorders of excessive somnolence scale (β = 0.200, 95% CI = 0.069–0.331, *p*_FDR_ = .011), compared to unexposed adolescents. There were no differences between the groups on the remaining sleep subscales.

**Table 2:** Effects of any prenatal alcohol exposure on parent‐reported sleep outcomes when adjusting for covariates and random effects

Parent‐reported sleep variables	β	95% CI	Unadjusted *p*	*p*_FDR_
Total sleep disorder score	0.517	0.127 to 0.906	.009**	.021*
Disorder of initiating and maintaining sleep	0.062	−0.131 to 0.255	.527	.604
Sleep breathing disorder	0.027	−0.026 to 0.080	.316	.442
Disorder of arousal	0.017	−0.016 to 0.051	.304	.442
Sleep–wake transition disorder	0.210	0.101 to 0.318	<.001***	.001**
Disorder of excessive somnolence	0.200	0.069 to 0.331	.003**	.011*
Sleep hyperhidrosis	0.012	−0.033 to 0.057	.604	.604

Unstandardised regression coefficients (β) and the associated 95% confidence intervals (95% CI), unadjusted *p*‐values (unadjusted *p*) and FDR adjusted *p* values (*p*_FDR_) for the effects of any prenatal alcohol exposure on parent‐reported sleep outcomes.

*p < .05, **p < .01, ***p < .001.

### Dose‐dependent associations

**Figure 1:**
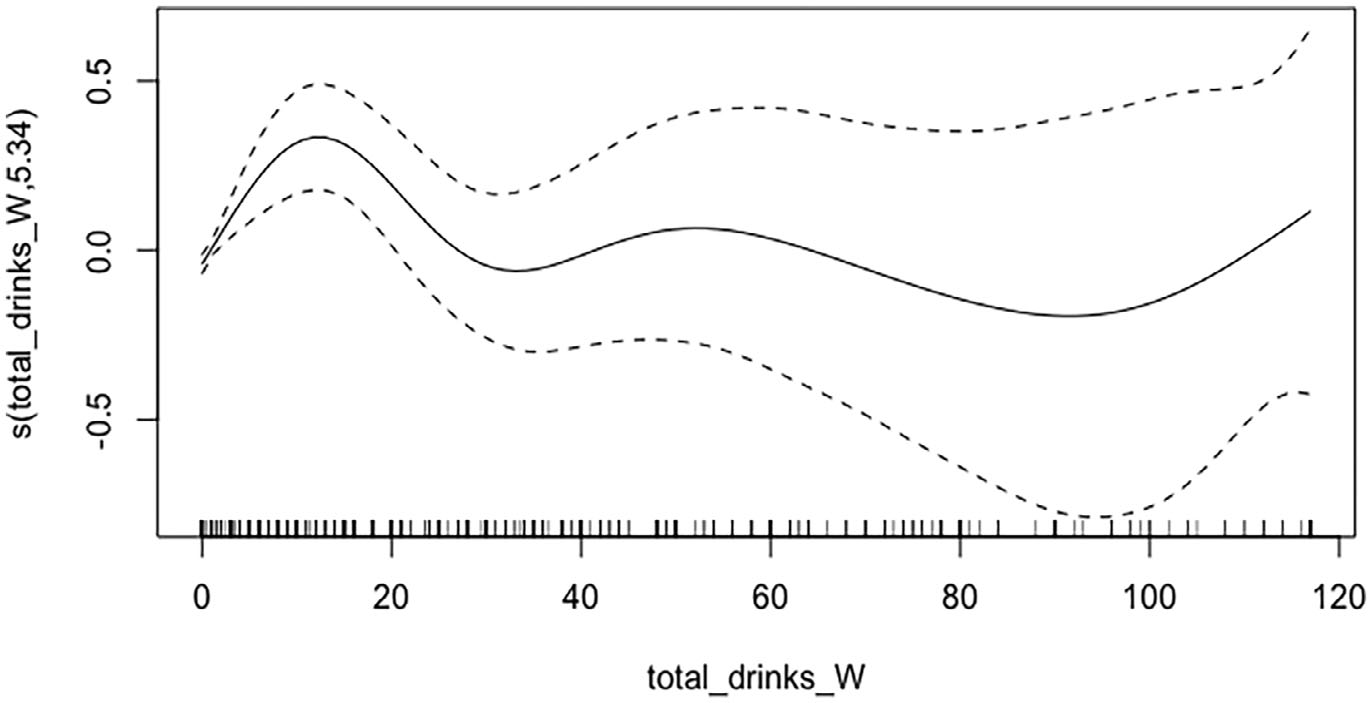
Generalised additive mixed model (GAMM) plot of the non‐linear association between total drinks and parent‐reported sleep–wake transition disorders, with the winsorised total drinks variable (total_drinks_W) presented on the *x*‐axis, and the smooth function for the winsorised total drinks variable on the link scale presented on the *y*‐axis, where s reflects the smooth term, total_drinks_W is the winsorised total drinks variable, and 5.34 is the effective degrees of freedom value

Table 3 presents the results from the seven GAMM models investigating the associations between the total number of drinks consumed during pregnancy (total drinks) and parent‐reported sleep disturbances. A statistically significant non‐linear association was observed between total drinks and sleep–wake transition disorders (EDF = 5.337, *F* = 3.047, *p*_FDR_ = .035). By inspecting the plot of this association (see Figure 1), we see that an increase in symptoms of sleep–wake transition disorder occurs predominantly with low‐level alcohol exposure (0–15 drinks). No dose‐dependent effects of PAE were observed for the remaining sleep disturbance outcomes.

**Table 3:** Dose dependent effects of prenatal alcohol exposure on parent‐reported sleep outcomes when adjusting for covariates and random effects

Parent‐reported sleep variables	EDF	*F* value	Unadjusted *p*	*p*_FDR_
Total sleep disorder score	4.275	1.512	.180	.420
Disorder of initiating and maintaining sleep	1.000	3.313	.068	.238
Sleep breathing disorder	1.565	0.296	.750	.750
Disorder of arousal	3.789	1.033	.450	.583
Sleep–wake transition disorder	5.337	3.047	.005**	.035*
Disorder of excessive somnolence	4.056	1.109	.395	.583
Sleep hyperhidrosis	1.628	1.685	.500	.583

Effective degrees of freedom (EDF), *F* values and associated unadjusted *p* values (unadjusted *p*) and FDR adjusted *p* values (*p*_FDR_) for the dose dependent effects of total prenatal alcohol exposure on parent‐reported sleep outcomes.

*p < .05, **p < .01, ***p < .001.

### PAE exposure pattern associations

Looking at the PAE exposure patterns, on average, women in the light stable group consumed 48.30 (*SD* = 31.23) drinks throughout pregnancy, those in the light reducing group consumed 15.65 (*SD* = 16.54) drinks, and those in the heavy reducing group consumed 39.66 (*SD* = 32.52) drinks. Participant characteristics for each group are provided in Table S3.

Results from the GLMMs adjusting for both covariates and random effects (see Table 4) showed that, compared to unexposed adolescents, the light reducing and heavy reducing PAE groups experienced worse overall parent‐reported sleep disturbances; however, after FDR correction, only the effect for the light reducing group remained statistically significant (β = 0.801, 95% CI = 0.285–1.317, *p*_FDR_ = .021). Regarding the parent‐reported sleep disturbance subscales, light reducing and heavy reducing groups experienced greater problems with sleep–wake transition disorders compared to those in the abstinent group. However, like with the overall sleep measure, only the effect for the light reducing group remained statistically significant after FDR correction (β = 0.296, 95% CI = 0.152–0.439, *p*_FDR_ = .002). All PAE groups experienced greater problems with excessive somnolence compared to those in the abstinent group; however, none survived FDR correction. No other differences were observed between exposure groups and unexposed adolescents on the sleep disturbance outcomes.

**Table 4:** Effects of prenatal alcohol exposure patterns on parent‐reported sleep outcomes when adjusting for covariates (including total drinks consumed) and random effects

Parent‐reported sleep variables	Exposure group	β	95% CI	Unadjusted *p*	*p*_FDR_
Total sleep disorder score	Light stable	1.912	−0.035 to 3.859	.054	.126
	Light reducers	0.801	0.285 to 1.317	.002**	.021*
	Heavy reducers	0.956	0.130 to 1.782	.024*	.087
Disorders of initiating and maintaining sleep	Light stable	0.483	−0.404 to 1.370	.284	.351
	Light reducers	0.160	−0.093 to 0.413	.214	.284
	Heavy reducers	0.279	−0.125 to 0.683	.175	.284
Sleep breathing disorder	Light stable	0.014	−0.225 to 0.252	.909	.909
	Light reducers	0.074	0.002 to 0.146	.044*	.112
	Heavy reducers	0.058	−0.056 to 0.173	.314	.366
Disorder of arousal	Light stable	0.039	−0.102 to 0.181	.584	.613
	Light reducers	0.030	−0.016 to 0.075	.199	.284
	Heavy reducers	0.033	−0.041 to 0.106	.380	.420
Sleep–wake transition disorder	Light stable	0.426	−0.165 to 1.017	.153	.284
	Light reducers	0.296	0.152 to 0.439	<.001***	.002**
	Heavy reducers	0.252	0.031 to 0.473	.025*	.088
Disorder of excessive somnolence	Light stable	0.770	0.181 to 1.359	.011*	.077
	Light reducers	0.212	0.030 to 0.394	.023*	.088
	Heavy reducers	0.300	0.009 to 0.591	.044*	.112
Sleep hyperhidrosis	Light stable	0.183	−0.023 to 0.389	.081	.168
	Light reducers	0.037	−0.021 to 0.096	.209	.284
	Heavy reducers	0.058	−0.034 to 0.150	.216	.284

Unstandardised regression coefficients (β) and the associated 95% confidence intervals (95% CI), unadjusted *p*‐values (unadjusted *p*) and FDR adjusted *p* values (*pFDR*) for the effects of PAE exposure patterns on parent‐reported sleep outcomes.

*p < .05, **p < .01, ***p < .001.

### Sensitivity analyses

#### *E*‐values

The *E*‐values for the statistically significant associations between binary PAE and sleep outcomes, and the patterns of PAE groups and parent‐reported sleep disturbance outcomes are reported in Table S4. *E*‐values ranged from 1.690 to 3.546, indicating that to fully account for the observed association, one or more unmeasured confounders would need to considerably increase the probability of an individual being prenatally exposed to alcohol and the probability of an individual experiencing higher overall disordered sleep, above and beyond the measured covariates, to account for the observed associations. Consider, for example, the association between the light reducing PAE group and the total summary score for all sleep disorders, which has an *E*‐value of 3.546. This suggests that one or more unmeasured confounders would need to more than triple both the probability of an individual being exposed to alcohol prenatally and the probability that they experience greater levels of sleep disorders to fully explain this association. Given the inclusion of several robustly measured covariates in the analyses, we anticipate the existence of unmeasured confounders of this magnitude to be unlikely, thus adding to the robustness of these findings.

#### Inverse probability weighting

The IPW adjustment was effective in emulating a more balanced sample, as indicated by the improved covariate balance shown in Figure S1. Results from the doubly robust models (see Table S5) aligned with those from the unweighted models in that adolescents with PAE experienced greater parent‐reported problems with overall sleep (β = 0.770, 95% CI = 0.260–1.281, *p*_FDR_ = .007), sleep–wake transitions (β = 0.274, 95% CI = 0.134–0.415, *p*_FDR_ <.001), and excessive somnolence (β = 0.249, 95% CI = 0.099–0.400, *p*_FDR_ = .004), compared to unexposed adolescents. There were no differences between the groups on the remaining sleep subscales. This indicates that the observed associations are not driven by differences in measured characteristics between groups.

#### Binary sleep outcomes

When conceptualised as a binary variable, those with any PAE had 16% higher odds of experiencing sleep breathing problems (aOR = 1.160, 95% CI = 1.048–1.285, *p*_FDR_ = .012; see Table S6). A dose‐dependent relationship was also observed between PAE and sleep breathing problems, with lower doses of PAE conferring the greatest impact; however, this association did not survive FDR correction (see Table S7 and Figure S2). Finally, those in the light‐reducing pattern of exposure had 23% higher odds of experiencing sleep breathing problems (aOR = 1.230, 95% CI = 1.079–1.403, *p*_FDR_ = .018), compared to those in the abstinent group (see Table S8). No other statistically significant associations were observed between PAE and the three dichotomised sleep outcomes.

## Discussion

There is an established literature linking both FASD and heavy PAE with poor sleep outcomes, particularly among infant and child populations (Alvik et al., 2011; Inkelis & Thomas, 2018; O'Rourke et al., 2024). However, less is known about how lower levels of PAE might also impact sleep, despite a growing consensus that even low levels of PAE can cause significant offspring harms (Lees et al., 2020). Moreover, it is unclear how PAE is associated with sleep in adolescent populations, despite the critical importance of sleep on adolescent development (Agostini & Centofanti, 2024; Tarokh et al., 2016). As such, this paper sought to address these gaps by investigating the associations between PAE, including low levels of PAE, and sleep in a large sample of adolescents.

As hypothesised, adolescents with PAE, compared to those without, experienced worse parent‐reported sleep outcomes. Although some studies in populations with FASD (O'Rourke et al., 2024) have identified associated disruptions across all sleep domains, our findings in this sample of primarily low to moderate PAE (26.47 drinks consumed across gestation) revealed the impact to be more localised, with sleep–wake transitions, excessive somnolence, and, in sensitivity analyses, sleep breathing disorders emerging as the domains most impacted by PAE. The robustness of these findings across rigorous sensitivity analyses underscores the reliability of these associations, highlighting that even low levels of PAE can have targeted and meaningful effects on sleep outcomes in adolescence, as reported by parents. While the effect sizes for statistically significant findings are quite small, it is important to note that this is not uncommon in ABCD data (Dick et al., 2021; Owens et al., 2021) and that while these small effects may not reach clinical thresholds, they are still relevant at a public health level, especially given the high prevalence of alcohol consumption during pregnancy in the population (Popova et al., 2017).

Sleep–wake transition disorders encompass any unusual or undesirable physical event or experience that occurs during the transition between sleep and wake states (e.g. sleep‐talking, twitching or jerking movements, vivid dreams). They tend to impact the quality of an individual's sleep, more so than the quantity, and people who experience problems with sleep–wake transitions often report fragmented, insufficient and poor‐quality sleep and a reduction in the restorative nature of their sleep (Bollu & Kaur, 2019; Medic, Wille, & Hemels, 2017). The negative impacts of sleep–wake transition disorders on sleep quality may underlie our finding that PAE is also associated with disorders of excessive somnolence, defined as an inability to stay awake, particularly during the day (Mayer, 2008). It is widely accepted that PAE alters an individual's neurobiology (Lebel, Roussotte, & Sowell, 2011; Lees et al., 2020), which may subsequently impact sleep. The field would benefit from further research investigating which brain regions or processes mediate the associations between PAE and problems with sleep–wake transitions and excessive somnolence specifically. Moreover, PAE is known to impact craniofacial morphology (Muggli et al., 2017) which in turn can contribute to the development of sleep breathing disorders (Flores‐Mir et al., 2013) and, as such, the increased incidence of sleep breathing disorders in those with PAE may be a direct consequence of such craniofacial morphology. However, given the association between PAE and sleep breathing disorders was only observed in the sensitivity analysis, these findings should be interpreted with caution and further research should work to establish the reliability of this link.

This study also sought to investigate the impact of dose and exposure pattern of PAE on sleep outcomes. Specifically, we hypothesised that a higher dose of PAE would lead to worse parent‐reported offspring sleep outcomes, however, this hypothesis was not supported, conflicting with existing research showing stronger associations between heavy PAE, rather than low‐moderate PAE, and sleep outcomes (Alvik et al., 2011; Inkelis & Thomas, 2018; Shang et al., 2006). In investigating the associations between patterns of PAE and sleep outcomes, our results showed an overall trend whereby those in the light reducing and heavy reducing exposure groups (i.e. those who were exposed to alcohol early in pregnancy prior to maternal knowledge of pregnancy) experienced worse sleep outcomes. However, only sleep–wake transition and sleep breathing problems among the light reducing group survived multiple comparison correction. This finding may indicate that, in the context of low to moderate PAE, the timing of the PAE is most impactful, with exposure early in pregnancy conferring the greatest risk for adverse sleep outcomes. This also aligns with previous research which identifies periods of rapid foetal development as being particularly susceptible to the impacts of PAE (May et al., 2013; Petrelli et al., 2018). However, it should also be noted that the light stable exposure group had the smallest sample size, which may have limited our ability to detect small to moderate effects.

There are several strengths and limitations of the current study which should be considered when interpreting these findings. Strengths include the use of the ABCD data that constitute a large, representative and well‐characterised sample. The ABCD Study also has a Retention Workgroup that monitors the study's enrolment and retention to ensure the sample maintains its diversity across, for example, race, socio‐economic and demographic factors (Ewing et al., 2022). The ABCD data also allowed us to conceptualise PAE in multiple ways, allowing for a nuanced examination of the impact of PAE on sleep in a sample closely representative of the general population. The present study also used sophisticated and robust analysis methods, including causal methods such as IPW. Regarding the limitations, although commonly used to document PAE (Lange et al., 2017), retrospective maternal report of alcohol consumption during pregnancy may be subject to reporting bias, largely due to the stigma associated with PAE, and recall bias (Lange et al., 2017). Similarly, although reliable in infant and child populations (Byars, Yolton, Rausch, Lanphear, & Beebe, 2012; Sadeh, Mindell, & Rivera, 2011), parental report of sleep problems in adolescence becomes less accurate (Fatima et al., 2016) and, as such, the reliability of the reported sleep problems may be low. This is further compounded by low internal consistency on two of the sleep subscales (sleep breathing disorder and disorders of arousal), which may have introduced measurement error and potentially attenuated associations with PAE. Thus, the adverse impact of lower levels of PAE on sleep may reflect a genuine effect, the effect of an uncontrolled variable that impacted offspring sleep, or limitations with the data itself. Replicating these analyses in other newly established large‐scale datasets, such as the Healthy Brain and Child Development (HBCD) Study (Volkow et al., 2024), would allow for several of the above limitations to be addressed and may lend further clarity. The HBCD Study is particularly well suited as it also intentionally oversamples for high‐risk pregnancies, including those exposed to substances prenatally. In addition to addressing the limitations mentioned above, future work should prioritise the development and evaluation of interventions aimed at improving sleep in populations with PAE.

In conclusion, this large, rigorous study was the first to examine the impact of PAE, including low levels of PAE, on parent‐reported sleep in adolescence. We found that PAE is associated with greater sleep disturbances, particularly sleep–wake transitions, excessive somnolence and sleep breathing. The results for the analyses investigating the role of PAE dose and pattern were contrary to what we expected, whereby lower levels of PAE were associated with greater sleep disturbances. We discussed how unique circumstances or characteristics in the light reducing PAE group might be driving these findings and explored the potential strengths and limitations of the study which should be considered in interpreting the results. Further research should consider addressing these limitations, for example, through objective measures of both PAE (e.g. detecting levels of PAE using baby teeth; Montag et al., 2022; Uban et al., 2018) and sleep (e.g. use of mobile and wearable technologies to track sleep; Bagot et al., 2018).

## Ethical considerations

The ABCD protocol was approved by the centralised institutional review board (IRB) at the University of California San Diego (#160091; most recent Continuing Review approval received 2 October 2025), and by the IRBs at the 21 data collection sites. Parents/caregivers provided signed informed consent and all youth participating gave assent.

**Key points**

**What is known?**

- Our study expands on existing research linking high levels of prenatal alcohol exposure (PAE) to poor sleep outcomes in children by investigating the effects of low‐to‐moderate PAE on parent‐reported adolescent sleep.

**What is new?**

- In this large and rigorous study, we identified three sleep domains affected by low‐to‐moderate PAE: sleep–wake transitions, excessive somnolence and sleep breathing problems.
- These findings have important implications in aiding the diagnosis and treatment of those with low‐to‐moderate PAE.

**What is relevant?**

- Given that sleep problems can exacerbate impairments associated with PAE, the development of effective sleep interventions to support adolescents with sleep problems resulting from PAE has the potential to substantially improve their quality of life.

## Supporting information


**Appendix S1.** Calculating the prenatal alcohol exposure (PAE) variables.
**Table S1.** Description of ABCD items used to calculate the prenatal alcohol exposure variables.
**Table S2.** Summary statistics for all variables used in the present study, using the observed data.
**Figure S1.** Covariate balance between PAE groups before and after weighting by the final weight.
**Table S3.** Summary statistics for all variables used in the present study, using the observed data, and split by pattern of PAE exposure.
**Table S4.** *E*‐values for the statistically significant associations between binary PAE and parent‐reported sleep outcomes, and the patterns of PAE groups and the parent‐reported sleep outcomes.
**Table S5.** Effects of any prenatal alcohol exposure on parent‐reported sleep outcomes when adjusting for covariates and random effects and including IPTW weights.
**Table S6.** Effects of any prenatal alcohol exposure on three parent‐reported sleep subscales (sleep breathing disorder, disorder of arousal, and sleep hyperhidrosis) when adjusting for covariates and random effects.
**Table S7.** Dose dependent effects of prenatal alcohol exposure on three parent‐reported sleep subscales (sleep breathing disorder, disorder of arousal, sleep hyperhidrosis) when adjusting for covariates and random effects.
**Figure S2.** GAMM plot of the non‐linear association between total drinks and parent‐reported sleep breathing disorders.
**Table S8.** Effects of prenatal alcohol exposure patterns on three parent‐reported sleep subscales (sleep breathing disorder, disorder of arousal and sleep hyperhidrosis) when adjusting for covariates (including total drinks consumed) and random effects.

## Data Availability

The data that support the findings of this study are available from Adolescent Brain Cognitive Development (ABCD) Study. Restrictions apply to the availability of these data, which were used under licence for this study. Data are available from https://nda.nih.gov/abcd/request‐access with the permission of Adolescent Brain Cognitive Development (ABCD) Study.
