# Supplementary material for: Associations between prenatal alcohol exposure and parent‐reported sleep disturbances in 10,336 adolescents: an Adolescent Brain Cognitive Development study
Source: J Child Psychol Psychiatry. 2026 May 21;67(10):1680–91. doi: 10.1111/jcpp.70165 (PMC13535761; doi:10.1111/jcpp.70165)
Supplement: Supplementary file 1 [file JCPP-67-1680-s001.docx]

**Associations between prenatal alcohol exposure and parent-reported sleep disturbances in 10,336 adolescents: An Adolescent Brain Cognitive Development (ABCD) Study**

**Supporting Information**

**Appendix S1. Calculating the prenatal alcohol exposure (PAE) variables**:

**PAE Variables**: PAE was measured at baseline using a version of the Developmental History Questionnaire (DHQ; Kessler et al., 2009; Merikangas et al., 2009) that was modified to include questions on maternal use of substances during pregnancy. These questions included ones asking about alcohol use before and after knowledge of pregnancy, the maximum number of drinks consumed on a single occasion before and after knowledge of pregnancy, and the average number of drinks consumed per week during pregnancy before and after knowledge of pregnancy. In addition to the modified DHQ, questions were also asked to assess how far along the biological mother was when they found out they were pregnant, whether the child was born prematurely, and if so, how many weeks premature they were. These additional questions were used alongside those from the modified DHQ to calculate the 3 PAE variables used in this study. Further information on these items can be found in Table S1.

**Table S1.** Description of ABCD items used to calculate the prenatal alcohol exposure variables.

| ABCD 5.1 Table Name | Variable name | Variable label | Response options |
| --- | --- | --- | --- |
| ph_p_dhx | devhx_8_alcohol | Before you/biological mom found out you/biological mom were pregnant, but while you might have been pregnant with this child, did you use any of the following? Alcohol? | Yes/no/don’t know |
| ph_p_dhx | devhx_9_alcohol | Once you/biological mom knew you/she were pregnant, were you/biological mom using any of the following? Alcohol? | Yes/no/don’t know |
| ph_p_dhx | devhx_8_alchohol_avg | Before knowledge of pregnancy what was the average number of drinks consumed per week? | Average number of drinks |
| ph_p_dhx | devhx_9_alchohol_avg | After knowing of pregnancy, what was the average number of drinks consumed per week? | Average number of drinks |
| ph_p_dhx | devhx_8_alchohol_max | Before knowing of pregnancy, what was the maximum number of drinks consumed in one sitting? | Number of drinks |
| ph_p_dhx | devhx_9_alchohol_max | After knowing of pregnancy, what was the maximum number of drinks consumed in one sitting? | Number of drinks |
| ph_p_dhx | devhx_7_p | How far along (in weeks) were you when you found out that you/biological mom were pregnant? | Number of weeks |
| ph_p_dhx | devhx_12a_p | Was the child born prematurely? | Yes/no/don’t know |
| ph_p_dhx | devhx_12_p | About how many weeks premature was the child when they were born? | 1-12, 13 = greater than 12, don’t know |

**Binary PAE**: Adolescents were categorised as having been exposed to alcohol prenatally if there was any parent-reported prenatal alcohol exposure either before or after knowledge of pregnancy. R code for the calculation of the binary PAE variable is shown below.

**Estimate of the total drinks consumed during pregnancy**: To examine the dose-dependent relationships between PAE and sleep outcomes, we calculated an estimate of the total number of drinks consumed during pregnancy following the method outlined by Lees et al. (2020). This was based on the following variables and formula, the R code for which is also provided below:

a) Average number of drinks consumed per week before pregnancy knowledge.
b) The week the mother found out they were pregnant. Two weeks were subtracted from reported week of pregnancy knowledge to adjust for conception date.
c) Average number of drinks consumed per week after pregnancy knowledge.
d) Gestational week of birth.

Estimate of total number of drinks consumed = a*b + c(d – b – 2)

**PAE exposure patterns**: According to established classifications (O’Leary et al., 2010), maternal drinking can be categorised as follows: i) abstinent (<1 standard drink/occasion throughout pregnancy); ii) light (1-2 drinks/occasions, <7 drinks/week); iii) moderate (3-4 occasions, <7drinks/week); iv) heavy (<5 drinks/occasion, 7+drinks/week); v) or binge drinking (5+ occasions) before and after knowledge of pregnancy. From these classifications, it is possible to calculate the following PAE exposure patterns (Lees et al., 2020):

1. Abstainers – abstinent throughout pregnancy
2. *Increasers – drank more after knowledge of pregnancy*
3. Light stable users – stable light alcohol consumption throughout pregnancy
4. Light reducers – light before knowing about pregnancy and abstinent after knowing of pregnancy
5. Heavier reducers:
   a. Heavier use to abstinence
   b. Heavier use to light use
6. *Heavy stable users – heavy alcohol use throughout pregnancy*

Due to small sample sizes, as was the case in Lees et al. (2020), the heavy stable users, and increasers were not included in this study.

The R syntax for the calculation of the PAE variables is available on GitHub (<https://github.com/emmakdevine/ABCD_Sleep_Paper>).

**Table S2.** Summary statistics for all variables used in the present study, using the observed data.

| Variable | N = 10,336^1^ | Unexposed Adolescents  N = 7,550^1^ | Adolescents with PAE N = 2,582^1^ | p-value^2^ |
| --- | --- | --- | --- | --- |
| **Prenatal Alcohol Exposure (PAE) variables^3^** | | | | |
| **PAE** |  |  |  |  |
| Unexposed adolescents | 7,550 (74.5%) | - | - | - |
| Exposed adolescents | 2,582 (25.5%) | - | - | - |
| Missing | 204^3^ | - | - |  |
| **Total drinks** | 6.50 (18.02) | 0.00 (0.00) | 26.47 (28.18) | <0.001 |
| Missing | 1,711 | 1,043 | 464 |  |
| **PAE patterns** |  |  |  | <0.001 |
| Abstainers | 7,175 (76.9%) | 7,172 (100.0%) | 3 (0.1%)^4^ |  |
| Light stable use | 94 (1.0%) | 0 (0.0%) | 94 (4.3%) |  |
| Light reducers | 1,278 (13.7%) | 0 (0.0%) | 1,278 (59.1%) |  |
| Heavy reducing | 787 (8.4%) | 0 (0.0%) | 787 (36.4%) |  |
| Missing | 1,002 | 378 | 420 |  |
| **Parent-reported sleep variables** | | | | |
| **Total** | 36.01 (7.89) | 35.70 (7.83) | 36.75 (7.84) | <0.001 |
| Missing | 268 | 188 | 76 |  |
| **Disorder of initiating and maintaining sleep** | 12.23 (3.94) | 12.15 (3.94) | 12.40 (3.90) | 0.005 |
| Missing | 268 | 188 | 76 |  |
| **Sleep breathing disorder** | 3.60 (1.07) | 3.60 (1.08) | 3.60 (1.02) | 0.786 |
| Missing | 268 | 188 | 76 |  |
| **Disorder of arousal** | 3.25 (0.65) | 3.25 (0.66) | 3.27 (0.64) | 0.111 |
| Missing | 267 | 188 | 75 |  |
| **Sleep-wake transition disorder** | 7.49 (2.18) | 7.39 (2.10) | 7.76 (2.32) | <0.001 |
| Missing | 268 | 188 | 76 |  |
| **Disorder of excessive somnolence** | 7.14 (2.63) | 7.04 (2.58) | 7.41 (2.71) | <0.001 |
| Missing | 266 | 188 | 74 |  |
| **Sleep hyperhidrosis** | 2.29 (0.89) | 2.28 (0.89) | 2.31 (0.89) | 0.136 |
| Missing | 268 | 188 | 76 |  |
| **Birth related variables** | |  |  |  |
| **Premature** |  |  |  | 0.009 |
| Yes | 1,952 (19.1%) | 1,481 (19.7%) | 443 (17.3%) |  |
| **Week of pregnancy knowledge** | 6.75 (6.49) | 6.71 (6.72) | 6.83 (5.71) | 0.424 |
| Missing | 1,143 | 711 | 251 |  |
| **Birth weight (lbs)** | 7.02 (1.48) | 7.00 (1.48) | 7.11 (1.43) | 0.001 |
| Missing | 1,030 | 702 | 218 |  |
| **Mother’s age at birth** | 29.65 (6.18) | 29.51 (6.20) | 30.23 (5.92) | <0.001 |
| Missing | 206 | 105 | 19 |  |
| **Prenatal tobacco exposure** | |  |  | <0.001 |
| Yes | 1,303 (12.9%) | 638 (8.5%) | 638 (25.1%) |  |
| Missing | 245 | 40 | 39 |  |
| **Prenatal cannabis exposure** | |  |  | <0.001 |
| Yes | 549 (5.5%) | 190 (2.5%) | 347 (13.8%) |  |
| Missing | 300 | 56 | 72 |  |
| **Prenatal cocaine exposure** | |  |  | <0.001 |
| Yes | 76 (0.8%) | 9 (0.1%) | 58 (2.3%) |  |
| Missing | 247 | 18 | 61 |  |
| **Prenatal heroin exposure** | |  |  | <0.001 |
| Yes | 18 (0.2%) | 5 (0.1%) | 10 (0.4%) |  |
| Missing | 261 | 17 | 72 |  |
| **Adolescent variables** | | | | |
| **Sex at birth** |  |  |  | 0.170 |
| Female | 4,909 (47.5%) | 3,547 (47.0%) | 1,254 (48.6%) |  |
| **Age** | 12.91 (0.65) | 12.91 (0.65) | 12.91 (0.65) | 0.695 |
| Missing | 1 | 0 | 0 |  |
| **Race ethnicity** |  |  |  | <0.001 |
| White | 5,592 (54.1%) | 3,873 (51.3%) | 1,656 (64.2%) |  |
| Black | 1,361 (13.2%) | 1,089 (14.4%) | 213 (8.3%) |  |
| Hispanic | 2,075 (20.1%) | 1,635 (21.7%) | 415 (16.1%) |  |
| Asian | 221 (2.1%) | 163 (2.2%) | 28 (1.1%) |  |
| Other | 1,086 (10.5%) | 790 (10.5%) | 269 (10.4%) |  |
| Missing | 1 | 0 | 1 |  |
| **Asthma** |  |  |  | 0.190 |
| Yes | 1,780 (17.2%) | 1,318 (17.5%) | 421 (16.3%) |  |
| **Brain injury** |  |  |  | 0.524 |
| Yes | 181 (1.8%) | 127 (1.7%) | 49 (1.9%) |  |
| **Cerebral palsy** |  |  |  | >0.999 |
| Yes | 15 (0.1%) | 10 (0.1%) | 4 (0.2%) |  |
| **Obesity** |  |  |  | 0.111 |
| Yes | 516 (5.0%) | 392 (5.2%) | 113 (4.4%) |  |
| **Bad life experiences** | 2.13 (2.09) | 2.08 (2.06) | 2.27 (2.14) | <0.001 |
| **Parent variables** |  |  |  |  |
| **Parental education** |  |  |  | <0.001 |
| < High school diploma | 590 (5.7%) | 531 (7.0%) | 50 (1.9%) |  |
| High school diploma or equivalent | 989 (9.6%) | 816 (10.8%) | 158 (6.1%) |  |
| College | 2,949 (28.6%) | 2,218 (29.4%) | 684 (26.5%) |  |
| Bachelor’s degree | 3,040 (29.4%) | 2,143 (28.4%) | 843 (32.6%) |  |
| Postgraduate degree | 2,756 (26.7%) | 1,831 (24.3%) | 847 (32.8%) |  |
| Missing | 12 | 11 | 0 |  |
| **Maternal depression** |  |  |  | <0.001 |
| Yes | 2,285 (23.3%) | 1,577 (21.6%) | 656 (27.0%) |  |
| Missing | 522 | 258 | 152 |  |
| **Paternal depression** |  |  |  | <0.001 |
| Yes | 1,397 (14.6%) | 944 (13.2%) | 424 (18.1%) |  |
| Missing | 759 | 396 | 235 |  |
| **Maternal anxiety** |  |  |  | 0.002 |
| Yes | 807 (8.2%) | 561 (7.6%) | 237 (9.7%) |  |
| Missing | 466 | 200 | 135 |  |
| **Paternal anxiety** |  |  |  | 0.214 |
| Yes | 478 (4.9%) | 342 (4.7%) | 129 (5.4%) |  |
| Missing | 576 | 271 | 173 |  |
| **Family conflict** | 2.07 (1.94) | 2.07 (1.91) | 2.07 (1.99) | 0.895 |
| Missing | 15 | 11 | 3 |  |
| **Caregiver acceptance** | 2.70 (0.38) | 2.70 (0.38) | 2.70 (0.38) | 0.695 |
| Missing | 12 | 9 | 3 |  |
| ^1^n (%); Mean (SD)  ^2^Welch Two Sample t-test; Pearson's Chi-squared test  ^3^204 participants were missing data on whether they were unexposed or exposed to alcohol during pregnancy. The summary statistics presented above for the Exposed and Unexposed Groups are excluding those 204 participants, while the summary statistics for the total sample column includes these participants.  ^4^Three participants reported having consumed alcohol before knowledge of pregnancy, 0 for the maximum number of drinks consuming on a single occasion before knowledge and pregnancy and the average number of drinks consumed per week before knowledge of pregnancy, resulting in 3 participants in the PAE group meeting criteria for the abstainers PAE Group. | | | | |

Table S3. Summary statistics for all variables used in the present study, using the observed data, and split by pattern of PAE exposure.

| **Variable** | **Abstainers** | **Light stable users** | **Light reducers** | **Heavy reducers** | **p-value^2^** |
| --- | --- | --- | --- | --- | --- |
|  | **N = 7,175^1^** | **N = 94^1^** | **N = 1,278^1^** | **N = 787^1^** |  |
| **Total drinks** | 0.00 (0.00) | 48.30 (31.23) | 15.65 (16.54) | 39.66 (32.52) | <0.001 |
| Missing | 665 | 7 | 72 | 49 |  |
| **Parent-reported sleep variables** | | | | | |
| **Disorder of initiating and maintaining sleep** | 12.14 (3.95) | 12.57 (4.11) | 12.24 (3.71) | 12.39 (3.95) | 0.245 |
| Missing | 178 | 1 | 32 | 30 |  |
| **Sleep breathing disorder** | 3.59 (1.08) | 3.41 (0.70) | 3.59 (0.96) | 3.59 (0.99) | 0.416 |
| Missing | 178 | 1 | 32 | 30 |  |
| **Disorder of arousal** | 3.25 (0.66) | 3.23 (0.49) | 3.26 (0.60) | 3.26 (0.67) | 0.933 |
| Missing | 178 | 1 | 32 | 29 |  |
| **Sleep-wake transition disorder** | 7.39 (2.11) | 7.80 (2.05) | 7.76 (2.36) | 7.72 (2.27) | <0.001 |
| Missing | 178 | 1 | 32 | 30 |  |
| **Disorder of excessive somnolence** | 7.02 (2.57) | 7.84 (2.84) | 7.27 (2.58) | 7.43 (2.74) | <0.001 |
| Missing | 178 | 1 | 32 | 28 |  |
| **Sleep hyperhidrosis** | 2.28 (0.88) | 2.39 (0.97) | 2.29 (0.85) | 2.29 (0.91) | 0.639 |
| Missing | 178 | 1 | 32 | 30 |  |
| **Total** | 35.66 (7.83) | 37.23 (7.37) | 36.41 (7.66) | 36.68 (7.64) | <0.001 |
| Missing | 178 | 1 | 32 | 30 |  |
| **Birth related variables** |  |  |  |  |  |
| **Premature** |  |  |  |  | 0.006 |
| Yes | 1,399 (19.6%) | 14 (15.1%) | 199 (15.6%) | 154 (19.7%) |  |
| Missing | 24 | 1 | 2 | 4 |  |
| **Week of pregnancy knowledge** | 6.73 (6.81) | 6.06 (5.58) | 6.66 (5.40) | 6.69 (4.94) | 0.792 |
| Missing | 638 | 6 | 70 | 48 |  |
| **Birth weight (lbs)** | 6.99 (1.48) | 7.42 (1.54) | 7.18 (1.35) | 7.05 (1.54) | <0.001 |
| Missing | 655 | 8 | 75 | 50 |  |
| **Mother’s age at birth** | 29.49 (6.21) | 33.71 (4.26) | 31.04 (5.50) | 29.46 (5.83) | <0.001 |
| Missing | 99 | 0 | 1 | 3 |  |
| **Prenatal tobacco exposure** |  |  |  |  | <0.001 |
| Yes | 599 (8.4%) | 7 (7.4%) | 168 (13.2%) | 282 (36.3%) |  |
| Missing | 10 | 0 | 3 | 10 |  |
| **Prenatal cannabis exposure** |  |  |  |  | <0.001 |
| Yes | 179 (2.5%) | 11 (11.8%) | 82 (6.5%) | 141 (18.3%) |  |
| Missing | 10 | 1 | 13 | 16 |  |
| **Prenatal cocaine exposure** |  |  |  |  | <0.001 |
| Yes | 8 (0.1%) | 1 (1.1%) | 6 (0.5%) | 10 (1.3%) |  |
| Missing | 3 | 0 | 1 | 7 |  |
| **Prenatal heroin exposure** |  |  |  |  | >0.999 |
| Yes | 5 (0.1%) | 0 (0.0%) | 0 (0.0%) | 0 (0.0%) |  |
| Missing | 3 | 1 | 1 | 3 |  |
| **Adolescent variables** |  |  |  |  |  |
| **Age** | 12.91 (0.65) | 12.86 (0.65) | 12.90 (0.65) | 12.92 (0.65) | 0.796 |
| **Sex at birth** |  |  |  |  | 0.164 |
| Female | 3,378 (47.1%) | 52 (55.3%) | 606 (47.4%) | 395 (50.2%) |  |
| **Race ethnicity** |  |  |  |  | <0.001 |
| White | 3,650 (50.9%) | 78 (83.0%) | 822 (64.3%) | 543 (69.1%) |  |
| Black | 1,055 (14.7%) | 2 (2.1%) | 111 (8.7%) | 42 (5.3%) |  |
| Hispanic | 1,572 (21.9%) | 11 (11.7%) | 191 (14.9%) | 130 (16.5%) |  |
| Asian | 162 (2.3%) | 0 (0.0%) | 22 (1.7%) | 4 (0.5%) |  |
| Other | 736 (10.3%) | 3 (3.2%) | 132 (10.3%) | 67 (8.5%) |  |
| Missing | 0 | 0 | 0 | 1 |  |
| **Obesity** |  |  |  |  | 0.019 |
| Yes | 382 (5.3%) | 0 (0.0%) | 53 (4.2%) | 37 (4.7%) |  |
| Missing | 7 | 0 | 1 | 1 |  |
| **Asthma** |  |  |  |  | 0.033 |
| Yes | 1,256 (17.5%) | 9 (9.6%) | 195 (15.3%) | 122 (15.5%) |  |
| **Brain injury** |  |  |  |  | 0.882 |
| Yes | 120 (1.7%) | 1 (1.1%) | 24 (1.9%) | 15 (1.9%) |  |
| **Cerebral palsy** |  |  |  |  | 0.673 |
| Yes | 10 (0.1%) | 0 (0.0%) | 3 (0.2%) | 1 (0.1%) |  |
| **Bad life experiences** | 2.08 (2.07) | 2.70 (2.17) | 2.10 (2.02) | 2.43 (2.21) | <0.001 |
| **Parent variables** |  |  |  |  |  |
| **Parental education** |  |  |  |  | <0.001 |
| < High school diploma | 524 (7.3%) | 0 (0.0%) | 15 (1.2%) | 11 (1.4%) |  |
| High school diploma or equivalent | 790 (11.0%) | 3 (3.2%) | 50 (3.9%) | 56 (7.1%) |  |
| College | 2,110 (29.4%) | 10 (10.6%) | 277 (21.7%) | 250 (31.8%) |  |
| Bachelor’s degree | 2,026 (28.3%) | 37 (39.4%) | 451 (35.3%) | 249 (31.6%) |  |
| Postgraduate degree | 1,715 (23.9%) | 44 (46.8%) | 485 (37.9%) | 221 (28.1%) |  |
| Missing | 10 | 0 | 0 | 0 |  |
| **Maternal depression** |  |  |  |  | <0.001 |
| Yes | 1,493 (21.5%) | 20 (22.0%) | 279 (22.7%) | 232 (31.1%) |  |
| Missing | 236 | 3 | 48 | 42 |  |
| **Paternal depression** |  |  |  |  | <0.001 |
| Yes | 902 (13.2%) | 17 (19.1%) | 195 (16.1%) | 150 (20.9%) |  |
| Missing | 359 | 5 | 70 | 70 |  |
| **Paternal anxiety** |  |  |  |  | 0.266 |
| Yes | 328 (4.7%) | 1 (1.1%) | 65 (5.3%) | 40 (5.4%) |  |
| Missing | 247 | 5 | 46 | 46 |  |
| **Maternal anxiety** |  |  |  |  | 0.002 |
| Yes | 525 (7.5%) | 5 (5.6%) | 92 (7.4%) | 89 (11.8%) |  |
| Missing | 182 | 5 | 34 | 31 |  |
| **Family conflict** | 2.07 (1.91) | 2.22 (1.90) | 1.94 (1.96) | 2.14 (2.00) | 0.066 |
| Missing | 11 | 0 | 2 | 1 |  |
| **Caregiver acceptance** | 2.70 (0.38) | 2.68 (0.44) | 2.72 (0.36) | 2.70 (0.39) | 0.439 |
| Missing | 9 | 0 | 2 | 1 |  |
| ^1^Mean (SD); n (%) | | | | | |
| ^2^One-way analysis of means; Fisher's Exact Test for Count Data with simulated p-values (based on 2000 replicates) | | | | | |

| **Table S4.** E-values for the statistically significant associations between binary PAE and parent-reported sleep outcomes, and the patterns of PAE groups and the parent-reported sleep outcomes. | | | | | | | | |
| --- | --- | --- | --- | --- | --- | --- | --- | --- |
|  | **PAE Binary** | | **PAE Exposure Groups** | | | | | |
|  |  |  | **Light stable** | | **Light reducer** | | **Heavy reducer** | |
| **Parent-reported sleep variables** | **E-value** | **Lower 95% CI** | **E-value** | **Lower 95% CI** | **E-value** | **Lower 95% CI** | **E-value** | **Lower 95% CI** |
| **Total sleep disorder score** | 2.580 | 1.495 | ^a^ | ^a^ | 3.546 | 1.923 | ^a^ | ^a^ |
| **Disorder of initiating and maintaining sleep** | ^a^ | ^a^ | ^a^ | ^a^ | ^a^ | ^a^ | ^a^ | ^a^ |
| **Sleep breathing disorder** | ^a^ | ^a^ | ^a^ | ^a^ | ^a^ | ^a^ | ^a^ | ^a^ |
| **Disorder of arousal** | ^a^ | ^a^ | ^a^ | ^a^ | ^a^ | ^a^ | ^a^ | ^a^ |
| **Sleep-wake transition disorder** | 1.715 | 1.422 | ^a^ | ^a^ | 1.945 | 1.563 | ^a^ | ^a^ |
| **Disorder of excessive somnolence** | 1.690 | 1.329 | ^a^ | ^a^ | ^a^ | ^a^ | ^a^ | ^a^ |
| **Sleep hyperhidrosis** | ^a^ | ^a^ | ^a^ | ^a^ | ^a^ | ^a^ | ^a^ | ^a^ |

E-values and the associated lower bound of the 95% confidence intervals (Lower 95% CI) for the statistically significant associations between binary PAE and parent-reported sleep outcomes, and the PAE groups and parent-reported sleep outcomes.

^a^ indicates associations that were not statistically significant at the *p*_FDR_ <0.05 level in the main analyses and thus no E-value was calculated.

**Figure S1.** Covariate balance between PAE groups before and after weighting by the final weight. This figure displays differences in means for the two exposure groups. Variables followed by an asterisk are unstandardised categorical variables.

| **Table S5.** Effects of any prenatal alcohol exposure on parent-reported sleep outcomes when adjusting for covariates and random effects and including IPTW weights. | | | | |
| --- | --- | --- | --- | --- |
| **Parent-reported sleep variables** | ***β*** | **95% CI** | **Unadjusted *p*** | ***pFDR*** |
| **Total sleep disorder score** | **0.770** | **0.260 – 1.281** | **0.003** | **0.007** |
| **Disorder of initiating and maintaining sleep** | 0.131 | -0.094 – 0.356 | 0.254 | 0.296 |
| **Sleep breathing disorder** | 0.053 | -0.017– 0.123 | 0.137 | 0.240 |
| **Disorder of arousal** | 0.027 | -0.017 – 0.070 | 0.225 | 0.296 |
| **Sleep-wake transition disorder** | **0.274** | **0.134 – 0.415** | **<0.001** | **<0.001** |
| **Disorder of excessive somnolence** | **0.249** | **0.099 – 0.400** | **0.001** | **0.004** |
| **Sleep hyperhidrosis** | 0.034 | -0.033 – 0.101 | 0.315 | 0.315 |

Unstandardised regression coefficients (*β*) and the associated 95% confidence intervals (95% CI), unadjusted p-values (unadjusted *p*) and FDR adjusted p values (*p****_FDR_***) for the effects of any prenatal alcohol exposure on parent-reported sleep outcomes with IPTW weights included in the analysis.
*<0.05, **<0.01, ***<0.001

| **Table S6.** Effects of any prenatal alcohol exposure on three parent-reported sleep subscales (sleep breathing disorder, disorder of arousal, and sleep hyperhidrosis) when adjusting for covariates and random effects. | | | | |
| --- | --- | --- | --- | --- |
| **Parent-reported sleep variables** | ***aOR*** | **95% CI** | **Unadjusted *p*** | ***pFDR*** |
| **Sleep breathing disorder** | **1.160** | **1.048 – 1.285** | **0.004** | **0.012** |
| **Disorder of arousal** | 1.093 | 0.960 – 1.244 | 0.180 | 0.180 |
| **Sleep hyperhidrosis** | 1.109 | 0.963 – 1.277 | 0.151 | 0.180 |

Adjusted odds ratios (aOR) and the associated 95% confidence intervals (95% CI), unadjusted p-values (unadjusted *p*) and FDR adjusted p values (***p_FDR_***) for the effects of any prenatal alcohol exposure on three parent-reported sleep subscales (sleep breathing disorder, disorder of arousal, sleep hyperhidrosis).
*<0.05, **<0.01, ***<0.001

| **Table S7.** Dose dependent effects of prenatal alcohol exposure on three parent-reported sleep subscales (sleep breathing disorder, disorder of arousal, sleep hyperhidrosis) when adjusting for covariates and random effects. | | | | |
| --- | --- | --- | --- | --- |
| **Parent-reported sleep variables** | **EDF** | **Chi^2^ Value** | **Unadjusted *p*** | ***pFDR*** |
| **Sleep breathing disorder** | **4.700** | **14.589** | **0.017** | 0.051 |
| **Disorder of arousal** | 3.818 | 5.032 | 0.454 | 0.454 |
| **Sleep hyperhidrosis** | 1.913 | 1.935 | 0.385 | 0.454 |

Effective degrees of freedom (EDF), Chi-squared values and associated unadjusted p values (unadjusted *p*) and FDR adjusted p values (*p*_FDR_) for the dose dependent effects of total prenatal alcohol exposure on three parent-reported sleep subscales (sleep breathing disorder, disorder of arousal, sleep hyperhidrosis).

*<0.05, ** <0.01, ***<0.001.

**Figure S2.** GAMM plot of the non-linear association between total drinks and parent-reported sleep breathing disorders, with the winsorised total drinks variable (total_drinks_W) presented on the x axis, and the smooth function for the winsorised total drinks variable on the link scale presented on the y-axis, where s reflects the smooth term, total_drinks_W is the winsorised total drinks variable, and 4.7 is the EDF value.

**Table S8.** Effects of prenatal alcohol exposure patterns on three parent-reported sleep subscales (sleep breathing disorder, disorder of arousal, and sleep hyperhidrosis) when adjusting for covariates (including total drinks consumed) and random effects.

| **Parent-reported sleep variables** | **Exposure group** | **aOR** | **95% CI** | **Unadjusted *p*** | ***pFDR*** |
| --- | --- | --- | --- | --- | --- |
| **Sleep breathing disorder** | | | | | |
|  | **Light stable** | 1.003 | 0.629 – 1.598 | 0.991 | 0.991 |
|  | **Light reducers** | **1.230** | **1.079 – 1.403** | **0.002** | **0.018** |
|  | **Heavy reducers** | 1.187 | 0.958 – 1.471 | 0.116 | 0.243 |
| **Disorder of arousal** | | | | | |
|  | **Light stable** | 1.358 | 0.758 – 2.412 | 0.230 | 0.296 |
|  | **Light reducers** | 1.191 | 0.999 – 1.418 | 0.051 | 0168 |
|  | **Heavy reducers** | 1.114 | 0.861 – 1.440 | 0.411 | 0.462 |
| **Sleep hyperhidrosis** | | | | | |
|  | **Light stable** | 1.833 | 0.984 – 3.413 | 0.056 | 0.168 |
|  | **Light reducers** | 1.156 | 0.956 – 1.399 | 0.135 | 0.243 |
|  | **Heavy reducers** | 1.205 | 0.902 – 1.608 | 0.206 | 0.296 |

Adjusted odds ratios (aORs) and the associated 95% confidence intervals (95% CI), unadjusted p-values (unadjusted *p*) and FDR adjusted p values (FDR *p*) for the effects of PAE exposure patterns on three parent-reported sleep subscales (sleep breathing disorder, disorder of arousal, and sleep hyperhidrosis).

*<0.05, **<0.01, ***<0.001
